# Evaluation of MDR1 and MRPA genes expression in different types of dry cutaneous leishmaniasis

**DOI:** 10.1186/s13104-019-4784-0

**Published:** 2019-12-12

**Authors:** Maryam Fekrisoofiabadi, Meisam Fekri, Alireza Moradabadi, Reza Vahidi, Morteza Khaleghi, Maryam Ram, Shahriar Dabiri

**Affiliations:** 1 Department of Pathology, Pathology and Stem Cell Research Center, Afzalipour Medical School, Kerman University of Medical Sciences, 22 Bahman Blvd, Kerman, Iran; 2 Department of Medicine, Montefiore New Rochelle Hospital, Albert Einstein College of Medicine, New York, USA; 3 Department of Hematology, School of Para Medicine, Arak University of Medical Science, Arak, Iran

**Keywords:** Multidrug-resistance 1, Multidrug-resistance protein A, Dry type cutaneous leishmaniasis, Real-time PCR

## Abstract

**Objective:**

The resistance to antimony-containing glucantime is a major obstacle to successful treatment, especially in endemic areas. Looking the molecular mechanisms involved in this drug resistance will help in choosing the best treatment. The aim of this study was to evaluate the expression of multidrug-resistance 1 (MDR1) and multidrug-resistance protein A (MRPA) genes in acute, chronic non-lupoid, and chronic lupoid forms of dry type cutaneous leishmaniasis (DTCL).

**Results:**

MDR1 gene was over-expressed as 14.4- and 1.56-folds in the chronic lupoid and acute forms compared with the chronic non-lupoid form, respectively. Results comparison showed P < 0.05 between the chronic non-lupoid and acute groups, P < 0.01 between acute and chronic lupoid groups, and P < 0.001 between the chronic non-lupoid and chronic lupoid groups. MRPA gene was over-expressed as 266 and 17.7-fold in the chronic lupoid and chronic non-lupoid forms compared with the acute form, respectively. Statistical analysis showed P < 0.01 between the chronic non-lupoid and chronic lupoid groups, P < 0.05 between acute and chronic non-lupoid groups, and P < 0.001 between the acute and chronic lupoid groups.

## Introduction

Leishmaniasis, one of the most common diseases among humans and animals [1], is caused by different species of protozoan parasites of the genus Leishmania, especially *Leishmania major* and *Leishmania tropica*. According to the World Health Organization (WHO), leishmaniasis is endemic in 88 countries, and 12 million people are infected by various forms of this pathogen, with an annual incidence of 1–1.5 million people [2]. From the histopathological point of view, acute, chronic lupoid, and chronic non-lupoid forms have special characteristics in terms of histiocyte and other inflammatory cells infiltration, presence of Leishman bodies and epithelioid granuloma [3–11]. Currently, quantitative real-time polymerase chain reaction (Real-time PCR) was developed for sensitive detection of different forms [12]. Also the FLASH-PCR used as a closed and sensitive method [13]. Considering the prevalence and complications of glucantime therapy in dry type cutaneous leishmaniasis (DTCL), the related drug resistance reports, and critical function of efflux pumps in resistance, the authors decided to use the Real-time PCR technique to determine the level of multidrug resistance protein 1 (MDR1) and multidrug-resistance protein A (MRPA) genes expression in acute, chronic non-lupoid, and chronic lupoid forms in paraffinized biopsy specimens.

## Main text

### Materials and methods

#### Sample collection

In this study, paraffinized skin biopsy samples from 90 positive *Leishmania tropica* patients, who referred (2010–2015) to the Dermatopathology Department of Afzalipour Medical School (Kerman University of Medical Sciences, Iran) and received at least three courses of glucantime, were applied. After staining of corresponding sections with Hematoxylin and Eosin, these human samples were classified into three forms: acute (n = 30), chronic non-lupoid (n = 30), and chronic lupoid (n = 30). It should be noted that skin samples came from different locations such as hand, lower lip, face, forearm, nose, and leg.

#### RNA extraction and cDNA synthesis

RNAs were extracted from paraffinized tissue samples using RNeasy FFPE kit (Qiagen, Germany) and corresponding cDNAs were made according to the RevertAid first strand cDNA kit (Thermo, k1622) protocol. To remove the DNA from RNA, the DNase I (Thermo) was used and finally the quantity and quality of resultant RNAs were evaluated using NanoDrop (ND-2000 Thermo, USA) and gel electrophoresis, respectively.

#### Primer design

In the present study, three sets of primer pairs corresponding to *Leishmania tropica* were designed using the VECTOR NTI Bioinformatic software and were then approved by NCBI with the PRIMER BLAST. These primers were synthesized by Korean Bioneer Company. The beta-actin gene (as a reference) from previous study was used to normalize the data [14]. The used primers for MDR1-F/R, MRPA-F/R, and beta-actin-F/R are shown in Table 1.

**Table 1:** The used primers in Real-time PCR assay

Primers	Sequences (5′–3′)
MDR1.F	5′-ATTGTCGCTTCTGGGGTTG-3′
MDR1.R	5′- ATCGTGTCGCTTGTGTCAC-3′
MRPA.F	5′-ATTACGTCCTGCAAGTCTGC-3′
MRPA.R	5′-ATTGTCGCTTCTGGGGTTG-3′
Beta actin.F	5′-ACCACCTTCAACTCCATCATG-3′
Beta actin.R	5′ -CTCCTTCTGCATCCTGTCG-3′

*MDR1* Multidrug resistance protein 1, *MRPA* Multidrug-resistance protein A, *F* Forward, *R* Reverse

#### Real-time PCR assay

To perform this assay, Gene Bio kit (Q9210) of Korea was used. For this purpose, 10 μL of Qmaster mix (2x) with SYBR Green, 5 μL of water, 1 μL of forward primer, 1 μL of reverse primer, 1 μL of ROX dye, and 2 μL of cDNA were mixed together. Amplification of desired fragments was performed using Applied Biosystem StepOne (ABI). The used program include: initial denaturation at 95 °C for 10 min (1 cycle), denaturation at 95 °C for 30 s, 66 °C for 30 s, and 72 °C for 30 s in 40 cycles. Using CT data (2^−ΔΔct^), the expression level of target genes was calculated in the three acute, chronic non-lupoid, and chronic lupoid forms of leishmaniasis. Finally the corresponding diagrams were drawn.

#### Statistical analysis

Using *t* test and by comparing mean of genes expression, the differences between experimental groups (two by two) were analyzed. P < 0.05 was considered as statistically significant.

### Results

**Fig. 1:**
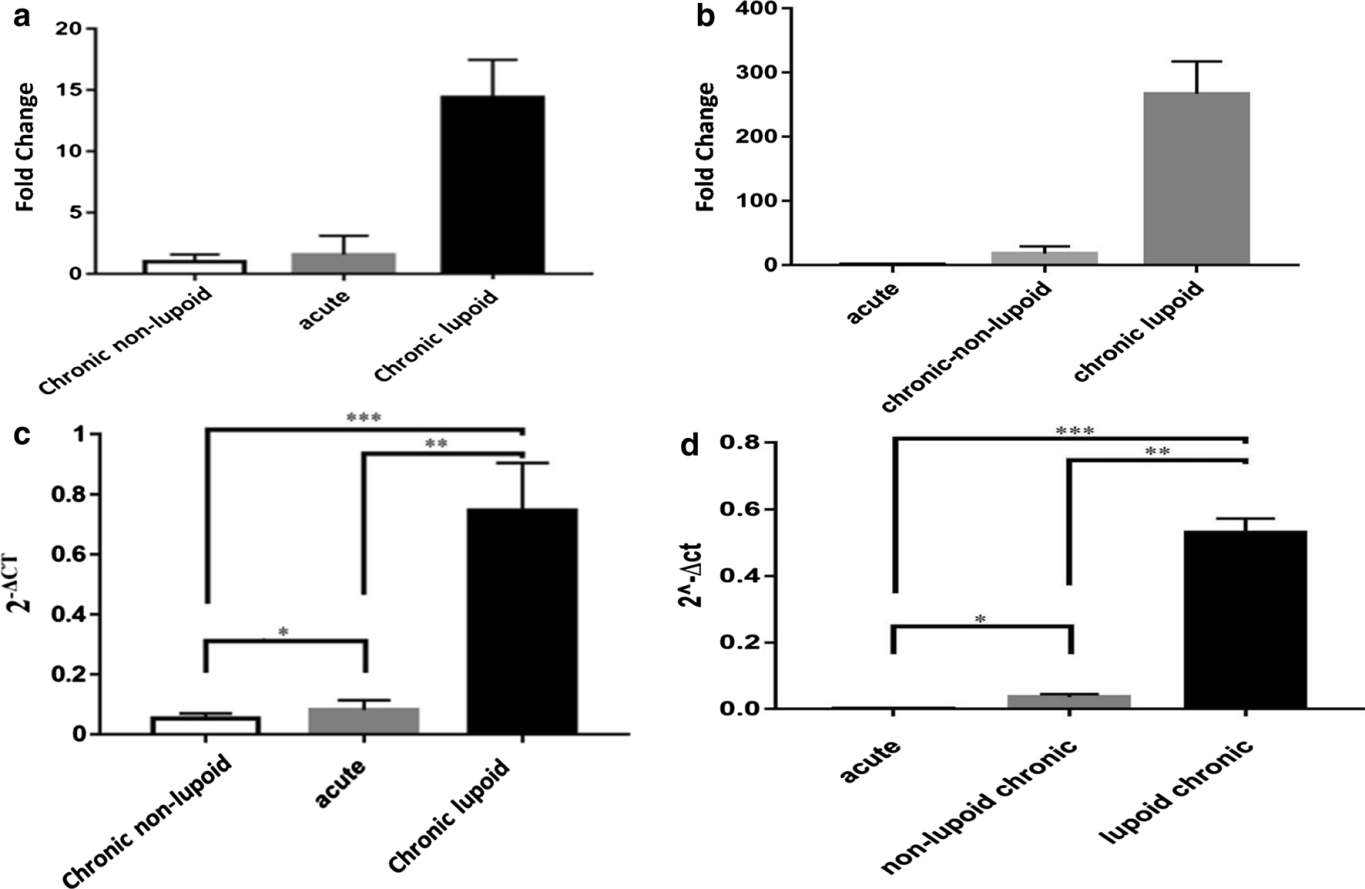
**a** The relative expression of MDR1 gene in the acute and chronic lupoid groups in compared with chronic non-lupoid were 1.56 and 14.4, respectively. **b** The relative expression of MRPA gene in the chronic non-lupoid and chronic lupoid groups in compared with acute group were 17.7 and 266, respectively. **c** Column chart of changes in the expression of MDR1 gene in acute, chronic non-lupoid, and chronic lupoid forms of *Leishmania tropica*. *, **, and *** show statistically significant difference between the chronic non-lupoid and acute groups (P < 0.05), between the acute and chronic lupoid groups (P < 0.01), and between the chronic non-lupoid and chronic lupoid groups (P < 0.001). **d** Column chart of changes in the expression of MRPA gene in acute, chronic non-lupoid, and chronic lupoid forms of *Leishmania tropica*. *, **, and *** show statistically significant difference between the chronic non-lupoid and acute groups (P < 0.05), between the acute and chronic lupoid groups (P < 0.001), and between the chronic non-lupoid and chronic lupoid groups (P < 0.01)

Real-time PCR method was used to determine the alterations of MDR1 and MRPA genes in parasitic tissues of acute, chronic non-lupoid, and chronic lupoid forms (30 paraffinized biopsy samples for each form) of *Leishmania tropica*. Compared with chronic non-lupoid specimens, the expression of MDR1 gene in acute and chronic lupoid paraffinized specimens were 1.56- and 14.4-folds, respectively. On the other hand, the expression of MRPA gene in chronic non-lupoid and chronic lupoid specimens were 17.7- and 266-folds, respectively, compared with acute specimens (Fig. 1a, b). As is clear, these differences were significant (Fig. 1c, d).

### Discussion

The control of *Leishmania tropica* is dependent on the immediate diagnosis and correct, timely, and effective treatment of patients. Despite some complications [15–18], chemotherapy, especially by pentavalent antimony compounds (like glucantime), is used more than other approaches. Limited information about underlying mechanisms has led to the emergence of resistance as a major factor affecting the treatment and control of cutaneous leishmaniasis, especially the urban type [19–21]. Due to the importance of drug resistance and high financial and medical costs for patients, the need to new treatments is felt. It is also necessary to identify resistant genes and find solutions to prevent their genetic effects. P-glycoprotein and Multidrug resistance-related protein (MRP), members of ATP-binding cassette (ABC) transporters family, are coded by the MDR1 and MRPA genes. Indeed, they are ATP-dependent drug efflux pumps that can reduce drug accumulation in resistant cells [2, 21–25]. Accordingly, the purpose of present study was to evaluate the expression of MDR1 and MRPA genes in acute, chronic non-lupoid, and chronic lupoid forms of Leishmaniasis using the Real-time PCR technique.

As presented in “Results” section, our findings showed that expression of MDR1 and MRPA in the chronic lupoid form was greater than chronic non-lupoid and acute forms. Contrary to our expectation, the level of MDR1 gene expression in the acute form was greater than chronic non-lupoid form (1.56-fold). Perhaps in this case, other genes and protein agents play a role in drug resistance. In line with this, 89 proteins were responsible for *Leishmania major* glucantime-resistance in a previous experiment [22]. For instance, the increased expression of ubiquitin and amino acid permease (AAP3) genes, increased activity of multidrug-resistance protein A (MRPA), protein tyrosine phosphatase (PTP), phosphoglycerate kinase (PGK), and the antagonists of the aquaglyceroporin (AQP1), mitogen activated protein kinase (MAPK), and calcineurin genes were probable causes of resistance. Similarly, Kazemi and co-workers identified antimony resistance markers in *Leishmania tropica* by cDNA-AFLP method [18].

The present work is the first study which compared the expression level of MDR1 and MRPA genes in acute, chronic non-lupoid, and chronic lupoid forms in the biopsy specimens of patients and also confirmed that the Real-time PCR method is a feasible method for investigating the resistance-related genes.

## Limitations

Accurately detection of MRPA and MDR1 in biopsy samples help to treat better in case with leishmaniosis. Using the paraffin-embedded biopsy samples make our samples collection time short. But it’s make the DNA extraction laboratories and effect on the quality of extracted DNA. In other hand to find better assumption of the resistance it’s better to evaluate other mechanisms other than the cytoplasmic channels.

## Data Availability

Please contact corresponding author (SD) for data requests.

## Abbreviations

MDR1: multidrug-resistance 1
MRPA: multidrug-resistance protein A
DTCL: dry type cutaneous leishmaniasis
AAP3: amino acid permease
PTP: protein tyrosine phosphatase
PGK: phosphoglycerate kinase
AQP1: antagonists of the aquaglyceroporin
MAPK: mitogen activated protein kinase
